# Medical Institute of Louisville

**Published:** 1841-09

**Authors:** 


### MEDICAL INSTITUTE OF LOUISVILLE.

We are happy to be able to announce that the prospects of the Institute, in reference to the approaching session, are, as far as we can discover, unclouded. Letters are constantly reaching the Professors, from distant parts of the West, asking for information; and indicating that although its fourth and last class numbered 208, and was the third in the Union, the Institute is as yet almost unknown in various parts of the country, whence in future it may expect liberal patronage. The last letters received from our colleague, Prof. Caldwell, announced that he was in good health, and had completed his purchases, embracing some articles of apparatus of the most splendid and useful kind, and valuable books, carefully selected from the bookstores of Paris, London, Dublin and Edinburgh, the whole of which have, indeed, reached the United States. Remaining in Europe long enough to attend the meeting of the British Scientific Association, he will be in Louisville in the month of October.

